# Admission Serum Albumin as a Predictor of Cardiovascular Complications in Non-Traumatic Intracerebral Hemorrhage

**DOI:** 10.3390/jcm15176840

**Published:** 2026-09-03

**Authors:** Amir Ghafarian, Hanieh Ghafarian, Anthony Liborio Corso, Fekade Tesfaye Danaso, Awni Shahait, Denise Battaglini, Faddi G. Saleh Velez, Adnan I. Qureshi, Andrea Loggini

**Affiliations:** 1 School of Medicine, International University of the Health Sciences, Basseterre P.O. Box 1267, Saint Kitts and Nevis; ghafarian.hanieh@gmail.com; 2 School of Medicine, St. George’s University, True Blue P.O. Box 7, Grenada; acorso@sgu.edu; 3 College of Medicine and Health Science, Arba Minch University, Arba Minch P.O. Box 21, Ethiopia; fekade.tesfaye7@gmail.com; 4 Division of Trauma and Acute Care Surgery, Southern Illinois Healthcare, Carbondale, IL 62901, USA; awni.shahait@sih.net; 5 School of Medicine, Southern Illinois University, Carbondale, IL 62901, USA; andrea.loggini@sih.net; 6 Anesthesia and Intensive Care, IRCCS Azienda Ospedaliera Metropolitana, 16149 Genova, Italy; denise.battaglini@unige.it; 7 Department of Surgical Sciences and Integrated Diagnostics, University of Genoa, 16132 Genoa, Italy; 8 Brain Stimulation and Neurorehabilitation Laboratory, Department of Neurology, University of Oklahoma Health Sciences Center, Oklahoma City, OK 73117, USA; faddi-salehvelez@ou.edu; 9 Zeenat Qureshi Stroke Institute, Department of Neurology, University of Missouri, Columbia, MO 65201, USA; qureshai@gmail.com; 10 Brain and Spine Institute, Southern Illinois Healthcare, Carbondale, IL 62901, USA

**Keywords:** intracerebral hemorrhage, serum albumin, hypoalbuminemia, cardiovascular complications

## Abstract

**Background:** Early cardiovascular complications after non-traumatic intracerebral hemorrhage (ICH) contribute to short-term morbidity and mortality, yet reliable prognostic markers remain limited. We examined the association between admission serum albumin and 30-day cardiovascular complications after ICH, including dose–response patterns and consistency across subgroups. **Methods:** In this retrospective cohort study using the TriNetX US Collaborative Network, adults hospitalized with ICH (ICD-10-CM I61) and serum albumin measured within 24 h of admission were stratified into reduced (≤3.4 g/dL) and normal (≥3.5 g/dL) groups and balanced by 1:1 propensity score matching on demographics, cardiovascular comorbidities, ICH severity, neurosurgical procedures, and medications. The primary outcome was a 30-day composite of all-cause death, acute myocardial infarction, heart failure, atrial fibrillation or flutter, ventricular arrhythmias, and Takotsubo cardiomyopathy. Sensitivity analyses addressed age, sex, comorbidity burden, and chronic hypoalbuminemia. **Results:** Of 241,228 patients, 94,925 (39%) had reduced albumin; 79,751 were retained per group after matching. The composite outcome occurred in 40.4% versus 33.3% (HR 1.28, 95% CI 1.26–1.30; *p* < 0.0001). Reduced albumin was associated with higher all-cause mortality (HR 1.50), myocardial infarction (HR 1.36), heart failure (HR 1.22), ventricular arrhythmias (HR 1.41), and Takotsubo cardiomyopathy (HR 1.41; all *p* < 0.005). Atrial fibrillation or flutter did not differ (HR 1.02; *p* = 0.779). A dose–response was observed, with severe hypoalbuminemia conferring greater composite risk (HR 1.49) than mild (HR 1.16). Findings were consistent across all sensitivity analyses. **Conclusions:** Reduced admission serum albumin is independently associated with higher short-term mortality and cardiovascular complications after ICH and may aid early risk stratification. Prospective studies are needed to clarify its utility.

## 1. Introduction

Non-traumatic intracerebral hemorrhage (ICH) accounts for approximately 28% of incident strokes worldwide, yet a disproportionate 46% of stroke-related deaths, reflecting a one-month case fatality of roughly 35% that has not improved over the past decade [1,2]. Acute management concentrates on hematoma expansion, cerebral edema, and intracranial pressure, yet a substantial proportion of early death and disability after ICH is cardiovascular rather than neurological in origin [3,4]. Newly diagnosed cardiac complications within weeks of ICH—myocardial injury, heart failure, arrhythmia, and stress cardiomyopathy—are common and independently worsen prognosis, but the patients who will develop them are difficult to identify at the time of admission [5,6].

The cardiac sequelae of acute brain injury are collectively termed stroke-heart syndrome and are attributed to a brain-heart axis in which sudden cerebral injury drives autonomic imbalance, catecholamine surge, systemic inflammation, and endothelial dysfunction, producing myocardial injury, ventricular dysfunction, and electrical instability [7,8,9]. Although characterized most fully after ischemic stroke, objective evidence of this axis exists in hemorrhage specifically: circulating cardiac troponin is elevated after intracerebral hemorrhage, experimental hemorrhage induces cardiac dysfunction in the absence of primary cardiac disease, and acute myocardial injury detected on serial troponin independently predicts major adverse cardiac events in this population [6,10,11]. Serum albumin is positioned within these same pathways; beyond maintaining oncotic pressure, it scavenges reactive oxygen species, binds inflammatory mediators, and stabilizes the endothelium, and reduced albumin marks adverse prognosis across cardiovascular disease and heart failure [12,13,14].

Two lines of ICH research have advanced in parallel without converging. Admission albumin, and the systemic inflammation it accompanies, has been examined in ICH as a predictor of infection, hematoma expansion, functional recovery, and mortality, but not of cardiovascular complications [15,16,17,18,19]. Conversely, the stroke-heart syndrome literature in ICH has quantified the incidence and downstream consequences of cardiac events without identifying a routinely available admission marker to flag patients at risk [5,20]. Where admission prediction of post-ICH risk has been pursued, it has largely relied on composite laboratory ratios incorporating fibrinogen, glucose, urea, or neutrophil counts, or on electrocardiographic and radiographic markers of myocardial injury, directed at mortality rather than a readily available marker of the cardiac phenotype [15,21,22,23,24]. The one analysis linking admission albumin to early cardiovascular complications was, moreover, performed in ischemic stroke, a condition whose hemostatic environment, inflammatory course, and arrhythmic profile differ from those of hemorrhage; whether the association transfers to the hemorrhagic population is unknown [20,25].

This study evaluated whether reduced admission serum albumin is associated with the 30-day risk of a composite of all-cause death and cardiovascular complications—acute myocardial infarction, heart failure, atrial fibrillation or flutter, ventricular arrhythmias, and Takotsubo syndrome—among adults hospitalized with non-traumatic ICH in a large federated health research network. Secondary aims were to resolve each component individually, to test for a dose–response gradient across the severity of hypoalbuminemia, and to determine whether any association persists across clinically relevant subgroups and in an analysis constructed to isolate acutely acquired hypoalbuminemia from chronic causes.

## 2. Materials and Methods

### 2.1. Study Design and Data Source

We conducted a retrospective observational cohort study using the TriNetX US Collaborative Network (TriNetX LLC, Cambridge, MA, USA; accessed on 30 June 2026), a federated health research network, which aggregates electronic medical records from approximately 80 healthcare organizations across the United States, encompassing roughly 80 million individuals. The network provides structured data across several domains, including demographic information, coded diagnoses (ICD-10-CM), laboratory measurements (LOINC), prescription medications (ATC classification), and procedural records (CPT). Data quality is maintained through ongoing automated assessment processes evaluating conformance, completeness, and plausibility with each database refresh. Reporting follows the Strengthening the Reporting of Observational Studies in Epidemiology (STROBE) guideline. The study design, exposure definition, outcome ascertainment, and sensitivity analysis framework were adapted from the methodology of Bucci et al., who applied an analogous propensity-matched TriNetX approach to examine albumin levels and early cardiovascular complications after acute ischemic stroke, and extended here to the ICH population [25].

This study utilized fully de-identified, pre-existing electronic health record data accessed through the TriNetX US Collaborative Network. In accordance with 45 CFR 46.104(d)(4), research conducted exclusively on pre-existing de-identified data is exempt from institutional review board (IRB) oversight. Accordingly, this study was exempt from IRB review, and no formal IRB approval or reference number was obtained. All procedures were conducted in accordance with the ethical principles of the Declaration of Helsinki. Individual informed consent was not required and was waived under the applicable federal exemption framework, as no patient-identifiable information was accessed at any stage.

### 2.2. Study Population

The study population consisted of adults aged 18 years or older with a primary hospital discharge diagnosis of ICH (ICD-10-CM I61) who had a serum albumin measurement (LOINC 1751-7) documented within 24 h of hospital admission. ICD-10-CM I61 defines non-traumatic intracerebral hemorrhage: it excludes traumatic intracranial hemorrhage but does not distinguish primary (spontaneous) from secondary non-traumatic causes, which cannot be identified from coded data [3]; the study population is therefore described as non-traumatic ICH throughout. The date of the recorded intracerebral hemorrhage diagnosis served as the index event date; all conditions documented prior to this date were treated as baseline characteristics. The study period was restricted to 1 January 2015 through 31 December 2025. Eighty healthcare organizations contributed data to this analysis.

Patients were stratified into two groups according to their admission albumin level: those with normal albumin (≥3.5 g/dL) and those with reduced albumin (≤3.4 g/dL). This threshold was selected on the basis of prior evidence linking albumin levels at or below 3.4 g/dL to increased cardiovascular risk and mortality in acutely hospitalized patients, and reflects the same validated cutoff applied in prior work on stroke and hypoalbuminemia [25,26,27]. The 24-h measurement window was chosen to capture albumin status during the acute phase of hemorrhagic injury.

### 2.3. Primary and Secondary Endpoints

The primary endpoint was the occurrence of a composite of cardiovascular events and all-cause mortality within 30 days of the index intracerebral hemorrhage, referred to hereafter as the composite outcome. This composite comprised all-cause death, acute myocardial infarction (I21, I21.0–I21.9), heart failure (I50, I50.1–I50.9), atrial fibrillation or flutter (I48, I48.0–I48.9), ventricular arrhythmias (ventricular tachycardia I47.2 and ventricular fibrillation I49.0), and Takotsubo cardiomyopathy (I51.81). All-cause death was retained within the composite because death is the dominant competing risk after ICH and its exclusion would bias estimates for the non-fatal components; the composite structure additionally mirrors that of Bucci et al. to preserve comparability with the ischemic stroke population [25]. Cause of death is not available as structured data in the network, precluding a cardiovascular-specific mortality endpoint. Takotsubo cardiomyopathy was included as a well-documented cardiac complication of acute cerebral injury that is distinct from heart failure in mechanism and course; its rarity does not materially influence the composite estimate, and it was additionally analyzed separately as a secondary endpoint, consistent with prior work in ischemic stroke [25]. Components were selected a priori as the recognized cardiac manifestations of stroke-heart syndrome reported after ICH [5,20,28]; conditions with inconsistent or nonspecific coding in this setting (troponin-defined myocardial injury, cardiac arrest) or with predominantly non-neurocardiac mechanisms after ICH (venous thromboembolism) were not included. Each individual component was additionally examined as a secondary endpoint. A complete list of ICD-10-CM codes used to ascertain outcomes is provided in Table S1.

### 2.4. Propensity Score Matching

The propensity score model incorporated the following covariates: age, sex, race and ethnicity, hypertensive diseases (I10–I16), ischemic heart disease (I20–I25), prior hemorrhagic stroke (I60–I62, pre-index), heart failure (I50), atrial fibrillation (I48), diabetes mellitus (E08–E13), chronic kidney disease (N18), dyslipidemia (E78), obesity (E65–E68), pulmonary embolism (I26–I28), peripheral arterial disease (I73.9), pneumonia (J12–J18), sepsis (A40–A41, R65.2), systemic connective tissue and autoimmune diseases (M30–M36), malnutrition (E40–E46), nephrotic syndrome (N04), liver cirrhosis (K74), inflammatory bowel disease including Crohn disease (K50) and ulcerative colitis (K51), burns (T20–T25), coma (R40.2–R40.24), cerebral edema (G93.6), brain herniation (G93.5), infratentorial hemorrhage location including cerebellar (I61.3) and brainstem (I61.6), cardiovascular procedures including electrocardiography (CPT 93000–93010), echocardiography (CPT 93303–93356), cardiac catheterization (CPT 93451–93572), external ventricular drain placement (CPT 61210), and surgical decompression or craniotomy (CPT 61312, 61314, 61315), and cardiovascular medications including anticoagulants (ATC B01A), antiplatelets (B01AC), beta-blockers (C07), antiarrhythmics (C01B), diuretics (C03), antilipemic agents (C10), calcium channel blockers (C08), and antianginals (C01D). Patients were also matched on ICH severity metrics including craniectomy/craniotomy for ICH (61312), GCS score at the index event (LOINC 9269-2), cerebral edema (G93.6), compression of brain (G93.5), coma (R40.2), and a composite of decompressive craniectomy/hematoma evacuation (CPT 1009097, 1009101, 1009104).

Race and ethnicity were included as matching covariates, and a female-restricted subgroup was prespecified, because both are established modifiers of the relationship between serum albumin and cardiovascular risk; ICH incidence and case fatality differ by sex and demographic group [2], and the albumin-cardiovascular association has been less consistently demonstrated in women than in men [29]. These categorizations were used solely to reduce confounding and to assess the consistency of the association, not to make inferences about any group.

### 2.5. Statistical Analysis

All analyses were carried out within the TriNetX online platform. Categorical variables were compared between groups using χ^2^ tests, and continuous variables using independent-sample *t* tests. To address confounding from the non-random distribution of albumin levels across patient subgroups, we applied 1:1 propensity score matching using a greedy nearest-neighbor algorithm based on logistic regression, with a caliper width of 0.1 pooled standard deviations. Balance was assessed using the absolute standardized mean difference, with values below 0.1 indicating adequate matching.

Time-to-event analyses in the matched cohorts were performed using Cox proportional hazard regression, with results expressed as hazard ratios and 95% confidence intervals. Survival distributions were visualized using Kaplan–Meier estimates and compared with the log-rank test over the 30-day follow-up. Patients were censored at the point they no longer contributed analyzable follow-up data. Adherence to the proportional hazards assumption was evaluated via scaled Schoenfeld residuals. A two-tailed significance threshold of *p* ≤ 0.05 was applied throughout.

### 2.6. Sensitivity Analyses and Subgroup Definitions

To account for the potential influence of non-stroke-related causes of hypoalbuminemia on the observed associations, five prespecified sensitivity analyses were performed. The first was restricted to patients older than 65 years, as albumin levels decline with advancing age [30]. The second included females only, given that the relationship between albumin and cardiovascular risk has been less consistently demonstrated in women [29]. The third was conducted in patients with hypertension and at least one of diabetes mellitus, chronic kidney disease, dyslipidemia, heart failure, or prior ischemic heart disease, to evaluate whether the association persists in a multimorbid population at elevated baseline risk [25]. The fourth examined a dose–response relationship by subdividing reduced albumin into mild (2.8–3.4 g/dL) and severe (≤2.7 g/dL) reduction, compared against the normal reference group. The fifth was restricted to patients without cirrhosis, malnutrition, nephrotic syndrome, inflammatory bowel disease, or autoimmune disease who additionally had a documented normal albumin (≥3.5 g/dL) at least one month before the index event, in the absence of sepsis, trauma, or burns, to exclude patients in whom hypoalbuminemia may have predated the ICH [25]. Because this subgroup excluded coded malnutrition and required a recent normal albumin, it additionally serves as a test of whether the association persists independent of baseline nutritional status.

During manuscript preparation, generative AI tools (Claude Fable 5.1, Anthropic, San Francisco, CA, USA) were used to assist with table and reference formatting; all content was reviewed, verified, and approved by the authors, who take full responsibility for the work.

## 3. Results

### 3.1. Cohort Characteristics

The initial cohort comprised 241,228 patients with intracerebral hemorrhage and an albumin measurement within 24 h of admission: 94,925 with reduced albumin (≤3.4 g/dL) and 146,303 with normal albumin (≥3.5 g/dL). Before propensity score matching, patients with reduced albumin had a substantially higher prevalence of hypertensive diseases, diabetes mellitus, ischemic heart disease, heart failure, chronic kidney disease, atrial fibrillation, sepsis, malnutrition, liver cirrhosis, coma, cerebral edema, and brain compression, as well as greater use of anticoagulants, beta-blockers, antiarrhythmics, calcium channel blockers, and diuretics (Table 1). After 1:1 propensity score matching, 79,751 patients were retained in each group. All covariates were well-balanced across the matched cohorts, with absolute standardized mean differences below 0.1 for the large majority of variables (Table 1).

### 3.2. Primary Outcome

**Figure 1:**
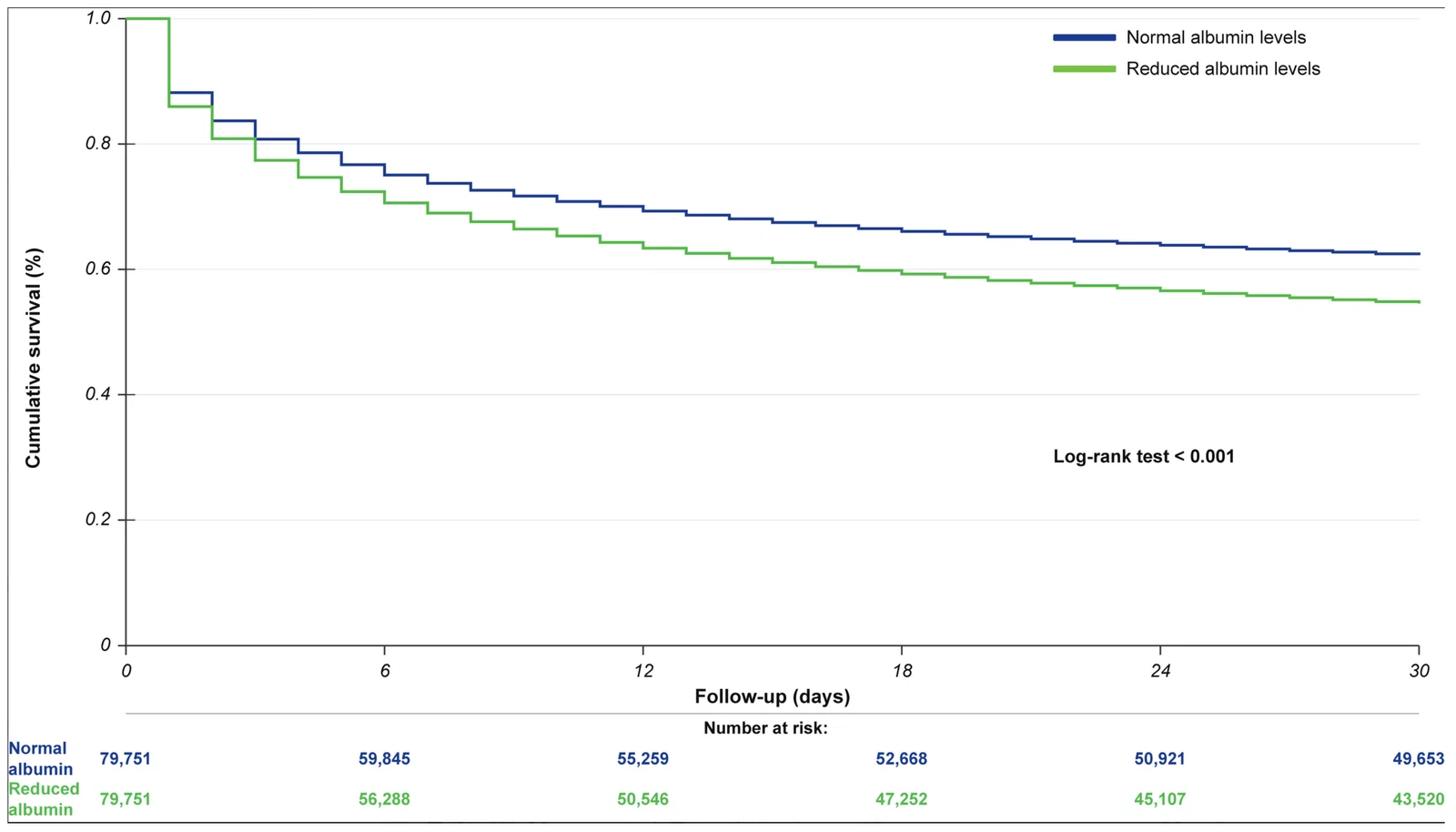
Thirty-day event-free survival for the composite of cardiovascular events and all-cause mortality by admission albumin status. Kaplan–Meier curves for the primary composite outcome (all-cause death, myocardial infarction, heart failure, atrial fibrillation/flutter, ventricular arrhythmias, or Takotsubo syndrome) over 30 days of follow-up in the 1:1 propensity-matched cohorts, comparing normal (≥3.5 g/dL, blue) and reduced (≤3.4 g/dL, green) admission serum albumin (log-rank *p* < 0.001). Numbers at risk are shown below the x-axis.

After propensity score matching, the primary composite outcome occurred in 32,233 patients with reduced albumin (40.4%) compared with 26,587 patients with normal albumin (33.3%), corresponding to a risk difference of 7.1% (95% CI 6.6–7.6%), a risk ratio of 1.21 (95% CI 1.20–1.23), and a hazard ratio of 1.28 (95% CI 1.26–1.30; *p* < 0.0001) (Table 2). Kaplan–Meier analysis demonstrated sustained separation in event-free survival beginning early in the follow-up period and persisting through 30 days, with survival probability at day 30 of 62.3% in the normal albumin group versus 54.6% in the reduced albumin group (log-rank *p* < 0.001; Figure 1).

### 3.3. Secondary Outcomes

**Figure 2:**
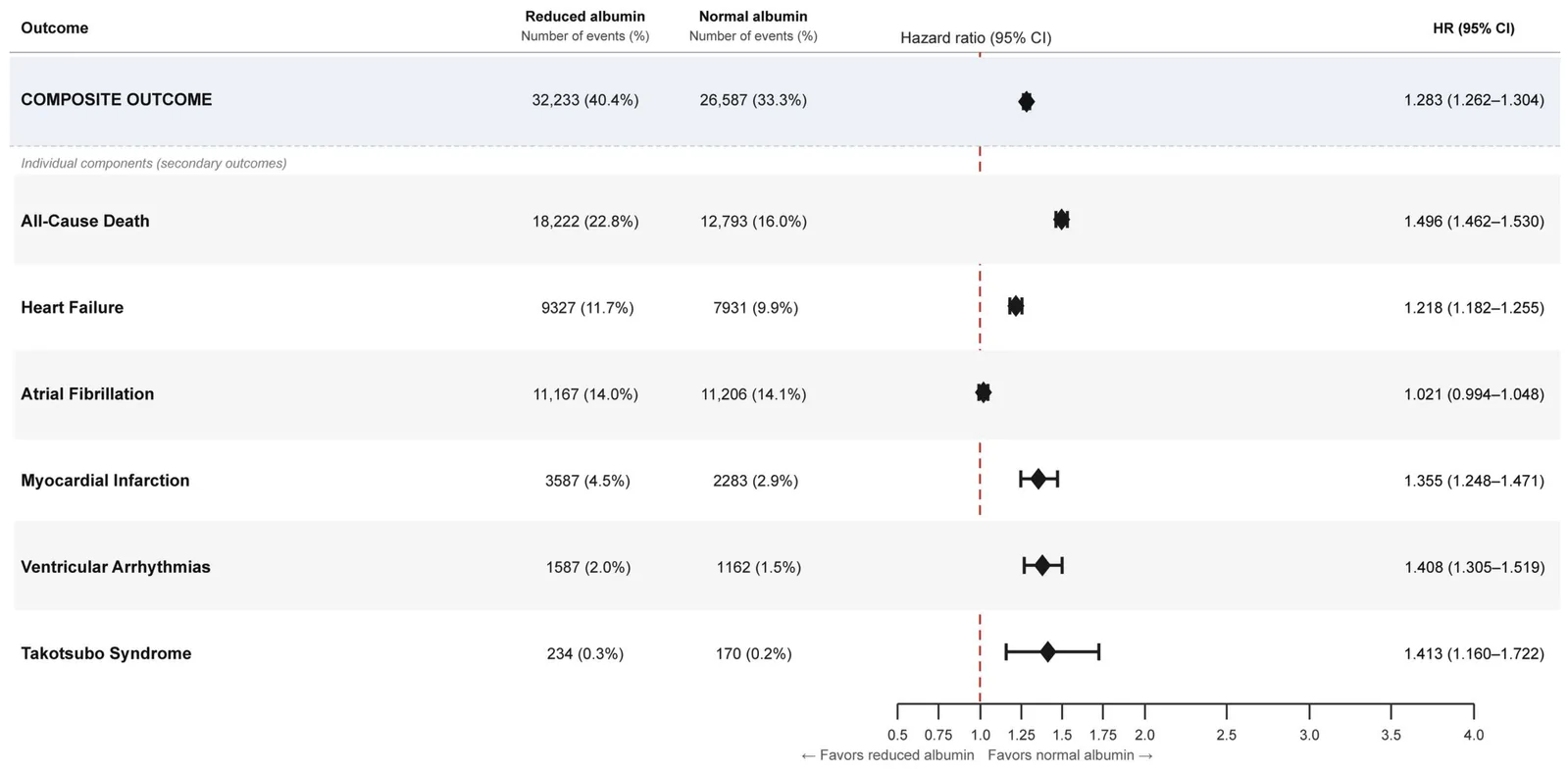
Hazard ratios for the composite outcome and individual components by albumin status. Forest plot showing event counts (%) in the reduced- and normal-albumin groups and hazard ratios with 95% confidence intervals for the 30-day composite outcome and each secondary component (all-cause death, heart failure, atrial fibrillation, myocardial infarction, ventricular arrhythmias, Takotsubo syndrome), comparing reduced versus normal admission serum albumin in the propensity-matched cohorts. Diamonds represent the point estimate of the hazard ratio for each outcome and the horizontal bars the corresponding 95% confidence intervals; marker size is uniform and does not encode statistical weight. The dashed vertical line marks a hazard ratio of 1.0, and the arrows beneath the axis indicate the direction of effect, with estimates to the right of the line favoring normal albumin.

The excess risk associated with reduced albumin was most pronounced for all-cause mortality, which occurred in 22.8% of patients with reduced albumin versus 16.0% with normal albumin (HR 1.50, 95% CI 1.46–1.53; *p* < 0.0001). Myocardial infarction was significantly more frequent in the reduced albumin group (4.5% versus 2.9%; HR 1.36, 95% CI 1.25–1.47; *p* < 0.0001), as were heart failure (11.7% versus 9.9%; HR 1.22, 95% CI 1.18–1.26; *p* < 0.0001), ventricular arrhythmias combining ventricular tachycardia and ventricular fibrillation (2.0% versus 1.5%; HR 1.41, 95% CI 1.31–1.52; *p* < 0.0001), and Takotsubo cardiomyopathy (0.3% versus 0.2%; HR 1.41, 95% CI 1.16–1.72; *p* = 0.0014). Atrial fibrillation and flutter occurred at statistically equivalent rates in both groups (14.0% versus 14.1%; HR 1.02, 95% CI 0.99–1.05; *p* = 0.779), representing the only component of the composite for which no significant association with albumin level was observed (Table 2; Figure 2).

### 3.4. Sensitivity Analyses

The association between reduced albumin and the primary composite outcome was consistent across all five prespecified sensitivity analyses (Table S3). Among patients older than 65 years, the hazard ratio for the composite outcome was 1.18 (95% CI 1.15–1.21), with significantly elevated risk of mortality (HR 1.39, 95% CI 1.34–1.43), myocardial infarction (HR 1.45, 95% CI 1.32–1.57), heart failure (HR 1.17, 95% CI 1.12–1.22), and ventricular arrhythmias (HR 1.24, 95% CI 1.10–1.40). Atrial fibrillation was not significantly elevated and trended slightly lower in the reduced albumin group in this subgroup (HR 0.965, 95% CI 0.932–0.999). Among females, the composite hazard ratio was 1.30 (95% CI 1.27–1.33), with consistent elevation across all components, including mortality (HR 1.42, 95% CI 1.37–1.47) and myocardial infarction (HR 1.59, 95% CI 1.46–1.71). In the multimorbid subgroup, results mirrored the main analysis for all outcomes except Takotsubo cardiomyopathy, which did not reach statistical significance (HR 0.975, 95% CI 0.725–1.310).

A dose–response relationship was observed between albumin level and cardiovascular risk. Patients with mild hypoalbuminemia (2.8–3.4 g/dL) had a composite hazard ratio of 1.16 (95% CI 1.14–1.19), whereas those with severe hypoalbuminemia (≤2.7 g/dL) had a substantially higher composite hazard ratio of 1.49 (95% CI 1.45–1.54). This gradient was most pronounced for all-cause mortality (mild: HR 1.37, 95% CI 1.33–1.41; severe: HR 1.95, 95% CI 1.88–2.02) and myocardial infarction (mild: HR 1.42, 95% CI 1.31–1.53; severe: HR 1.83, 95% CI 1.69–1.97). Atrial fibrillation remained non-significant across both albumin strata.

In the analysis restricted to patients with acutely acquired hypoalbuminemia (baseline characteristics before and after matching are shown in Table S2), excluding those with chronic conditions known to lower albumin and requiring a documented normal albumin at least one month before the index event, reduced albumin remained significantly associated with the composite outcome (HR 1.26, 95% CI 1.23–1.29) and with all individual components except Takotsubo cardiomyopathy, which did not reach statistical significance in this subgroup (HR 1.31, 95% CI 0.94–1.82), and atrial fibrillation, which showed a nominally lower event rate in the reduced albumin group (HR 0.934, 95% CI 0.898–0.971).

## 4. Discussion

### 4.1. Principal Findings

In this large multicenter propensity-matched cohort study of patients with ICH, reduced serum albumin measured within 24 h of admission was independently associated with a significantly higher 30-day risk of death and major cardiovascular complications. Patients with hypoalbuminemia experienced a 28% higher hazard of the composite endpoint and a 50% higher hazard of all-cause mortality than patients with normal albumin. Reduced albumin was also associated with higher risks of myocardial infarction, heart failure, ventricular arrhythmias, and Takotsubo cardiomyopathy, whereas no association was observed for atrial fibrillation or flutter. All-cause mortality was the largest single contributor to the composite. The cardiovascular specificity of the association therefore rests on the component-level estimates, in which myocardial infarction, heart failure, ventricular arrhythmias, and Takotsubo cardiomyopathy each remained independently associated with reduced albumin. A dose–response relationship, with progressively higher risk among patients with severe hypoalbuminemia, and consistency across older adults, women, multimorbid patients, and a subgroup isolating acute hypoalbuminemia strengthen the biological plausibility of these findings. The latter subgroup, by excluding coded malnutrition and requiring a documented normal albumin before the hemorrhage, also indicates that the association is not attributable to pre-existing nutritional depletion.

### 4.2. Contextualization Within the Literature

These findings extend the stroke-heart syndrome literature into the hemorrhagic stroke population. Prior ICH studies have shown that the hyperacute phase can precipitate cardiac complications through neurogenic myocardial injury, autonomic dysregulation, and systemic inflammatory activation, and that such complications are common and prognostically important [20,28,31]. Separately, hypoalbuminemia after ICH has been shown to predict mortality, infection, hematoma expansion, and poor neurological recovery [24,32,33]. The present analysis links these two previously separate lines of investigation by demonstrating that reduced admission albumin also identifies patients at higher early cardiovascular risk. The framework relating admission albumin to early cardiovascular complications had previously been established in ischemic stroke [25]; because intracerebral hemorrhage differs from ischemic stroke in its hemostatic environment, inflammatory trajectory, and arrhythmic profile, the extension of this association to the hemorrhagic population is not assured a priori and is the principal contribution of this work.

The absence of an association with atrial fibrillation is consistent with, rather than contrary to, existing hemorrhagic-stroke evidence. Pooled analyses of cardiac complications after ICH have reported that atrial fibrillation or flutter occurs less frequently after intracerebral hemorrhage than after ischemic stroke, whereas non-atrial cardiac events occur at comparable rates [5,28]. Within this context, the null atrial-fibrillation finding suggests that reduced albumin tracks ischemic and ventricular myocardial injury and pump dysfunction more closely than atrial electrical remodeling in the hemorrhagic population, although this interpretation requires confirmation.

### 4.3. Biological and Clinical Mechanisms

Several mechanisms may underlie these associations. Acute ICH triggers substantial sympathetic activation through disruption of central autonomic pathways, producing catecholamine excess, myocardial oxygen supply-demand mismatch, endothelial dysfunction, and direct cardiomyocyte injury [8,34]. Objective evidence that this axis produces measurable myocardial damage after hemorrhage specifically comes from biomarker studies reporting elevated cardiac troponin following brain hemorrhage [15]. Hypoalbuminemia may amplify these effects: albumin scavenges reactive oxygen species, binds inflammatory mediators, and preserves endothelial integrity, so lower levels may reflect diminished physiologic reserve during acute systemic stress [16,35,36]. The interplay among inflammation, nutrition, and albumin in acute brain injury is bidirectional. Albumin behaves as a negative acute-phase reactant: systemic inflammation after hemorrhage suppresses hepatic synthesis, increases capillary permeability with transcapillary escape of albumin, and accelerates protein catabolism, so serum levels can fall within hours of injury independent of nutritional intake [17]. Malnutrition, where present, amplifies the inflammatory response, impairs immune competence and tissue repair, and raises the risk of infectious complications, which in turn deepen the inflammatory suppression of albumin [18]. This cycle implies that admission hypoalbuminemia integrates systemic inflammatory burden and overall disease severity rather than nutritional status alone, a reading consistent with the rapid decline observed in the acute phase and with the persistence of the association in the subgroup without chronic albumin-lowering conditions. Hypoalbuminemia has additionally been associated with endothelial dysfunction, hypercoagulability, altered drug pharmacokinetics, and impaired microvascular perfusion, which may increase susceptibility to myocardial injury and circulatory instability [37]. Low albumin is likewise a recognized marker of poor prognosis in heart failure, irrespective of ejection fraction [11]. Alternatively, reduced albumin may function as an integrated marker of illness severity, reflecting systemic inflammation, neuroendocrine stress, malnutrition, frailty, or occult comorbidity burden not fully captured in administrative datasets [27]. Acute kidney injury illustrates this pathway concretely: it occurs in approximately 15% of patients after ICH and is associated with substantially higher mortality [38], hypoalbuminemia independently predicts both the development of AKI and death following it [39], and yet AKI was not captured among the study outcomes; although baseline chronic kidney disease was balanced by matching, incident post-index AKI remains an unmeasured factor that may have contributed to the mortality difference observed. The prognostic relevance of nutritional status extends beyond ICH, as nutritional deficits impair recovery across a range of acute injuries [40].

### 4.4. Statement of Novelty

This study makes several concrete contributions. First, to our knowledge, it is the first to evaluate admission serum albumin as a predictor of early cardiovascular complications in the hemorrhagic stroke population specifically, a condition mechanistically distinct from the ischemic stroke setting in which this association was previously described. Second, its scale—approximately 80 U.S. healthcare organizations and 79,751 matched patients per group—permitted stable estimation of relatively uncommon endpoints such as Takotsubo cardiomyopathy and ventricular arrhythmias that smaller ICH cohorts cannot support. Third, it demonstrates a graded dose–response across the severity of hypoalbuminemia. Fourth, it identifies a hemorrhage-specific pattern in which albumin is associated with ischemic and ventricular events and death but not with atrial fibrillation. Finally, a prespecified subgroup requiring documented normal albumin at least one month before the event isolates acutely acquired hypoalbuminemia, addressing a confounder that most prior albumin-ICH studies could not.

### 4.5. Clinical Implications and Generalizability

Serum albumin is inexpensive, universally available, and routinely measured during hospitalization, making it a potentially useful risk-stratification tool in ICH. Early identification of patients at elevated cardiovascular risk may support closer telemetry monitoring, lower thresholds for cardiac biomarker assessment, echocardiographic evaluation in selected patients, and more proactive collaboration among neurocritical care, stroke, and cardiology teams [41]. These findings should not be interpreted as evidence that correcting hypoalbuminemia improves outcomes. Trials of albumin administration in acute neurologic injury have produced mixed and at times unfavorable results: a meta-analysis of randomized trials found no mortality benefit from albumin in critically ill patients [42]; the SAFE trial reported worse outcomes with albumin-based resuscitation in traumatic brain injury [43]; and the ALIAS part 2 trial in acute ischemic stroke found no benefit and increased pulmonary edema [44]. The ACHIEVE trial, evaluating albumin within 24 h of ICH, was terminated early for poor enrollment, leaving the role of exogenous albumin in ICH untested in a completed prospective trial [45]. These findings therefore generalize to the identification of higher-risk patients through a routinely available marker, and do not establish albumin as a modifiable therapeutic target. The cohort was drawn from patients with an early albumin measurement at participating U.S. institutions and may not generalize to patients without such contact or to non-U.S. settings.

### 4.6. Limitations

Several limitations warrant consideration. First, the retrospective observational design is subject to unmeasured confounding; although propensity score matching incorporated an extensive covariate set, residual confounding from variables not captured in TriNetX cannot be excluded, and would bias the association in an uncertain direction. Second, as a federated network, TriNetX is subject to entry errors and variable completeness across institutions, and coding accuracy depends on institutional documentation, introducing possible misclassification of exposures and outcomes; because a coded outcome may be absent when an event occurred, event rates may be underestimated, biasing associations toward the null. In addition, I61 does not distinguish primary from secondary non-traumatic hemorrhage, and coded data cannot confirm the absence of an underlying structural source such as a vascular malformation or hemorrhage-prone neoplasm; the proportion of such cases and their distribution across the matched cohorts are unknown, and the direction of any resulting bias cannot be determined. Because inclusion required an early albumin measurement, the cohort was limited to patients with sufficient healthcare contact and may differ from the broader ICH population, limiting generalizability. Third, although the model incorporated admission Glasgow Coma Scale score (LOINC 9269-2) and additional diagnostic and procedural surrogates of severity, GCS was not uniformly recorded, and the platform does not capture hematoma volume or laterality, intraventricular extension, baseline NIHSS, the ICH score, or post-index characteristics such as hemorrhagic expansion or neurological deterioration. The available surrogates are coded diagnoses and procedural records, and neurosurgical procedures in particular may reflect treatment selection and institutional practice patterns as well as disease severity, so substantial residual confounding by hemorrhage severity cannot be excluded. Fourth, outcomes occurring after discharge or outside the network may be inconsistently captured, potentially underestimating 30-day event rates. Fifth, individual-level socioeconomic indicators are not consistently available and could not be incorporated. Sixth, although the final sensitivity analysis was designed to isolate acutely acquired hypoalbuminemia, subclinical or intermittent hypoalbuminemia may have preceded the hemorrhage in some patients. Seventh, because the composite incorporated all-cause death, and death precludes ascertainment of subsequent non-fatal events, mortality functions as a competing risk for the individual non-fatal components; the reported hazard ratios for those components are cause-specific hazards estimated by treating competing death as censoring. Because early mortality was higher in the reduced-albumin group, this approach may overestimate the cumulative incidence of non-fatal components in that group and bias their cause-specific hazard ratios away from the null, whereas the composite outcome and all-cause mortality are unaffected because death is an event rather than a competing risk in those analyses; subdistribution-hazard (Fine-Gray) modeling would be required to account formally for competing mortality, and the non-fatal estimates should be interpreted accordingly. Eighth, cause of death is not captured as structured data in the network; the composite therefore incorporates all-cause rather than cardiovascular death, and deaths from non-cardiovascular causes (sepsis, neurological deterioration, withdrawal of care) contribute to the composite estimate. The component analyses of non-fatal outcomes mitigate this in part. Ninth, the platform does not support continuous-exposure or spline modeling, so albumin was analyzed categorically using validated thresholds; while the three-tier analysis demonstrates a monotonic gradient, the precise shape of the albumin-risk relationship could not be characterized. Similarly, admission blood pressure is not reliably captured as structured data across contributing institutions and could not be incorporated; although diagnosed hypertensive disease was matched and severity surrogates partially index the downstream consequences of acute hypertension, residual confounding by admission hemodynamics cannot be excluded. Finally, the platform does not provide granular functional outcomes, neurological disability scores, or patient-reported outcomes, limiting broader clinical conclusions.

### 4.7. Future Directions

Two lines of investigation follow directly. A prospective ICH cohort could test whether adding admission albumin to established prognostic scores improves discrimination for 30-day cardiovascular events, and whether combining albumin with troponin and natriuretic peptides identifies a higher-risk phenotype than any marker alone. Such a cohort would also permit modeling albumin as a continuous variable with restricted cubic splines, defining the shape of the exposure-risk relationship and any threshold effects that categorical analysis cannot resolve. Separately, a study applying subdistribution-hazard (Fine-Gray) modeling in a competing-risk framework could quantify the cumulative incidence of individual non-fatal cardiac events after ICH while accounting for the high early mortality of the hypoalbuminemic group, clarifying whether the component-level associations observed here reflect true differences in event risk.

## 5. Conclusions

Among patients hospitalized with non-traumatic ICH, reduced serum albumin measured within 24 h of admission was independently associated with higher 30-day mortality and early cardiovascular complications, including myocardial infarction, heart failure, ventricular arrhythmias, and Takotsubo cardiomyopathy, but not atrial fibrillation. Admission hypoalbuminemia may serve as a readily available early marker of susceptibility to stroke-heart syndrome in the hemorrhagic stroke population. Prospective studies are needed to validate these findings, refine the prognostic utility of albumin in ICH risk stratification, and determine whether hypoalbuminemia represents a modifiable target or a marker of systemic vulnerability.

## Figures and Tables

**Table 1:** Baseline Characteristics of Study Cohorts Before and After Propensity Score Matching.

	Before Propensity Score Matching				After Propensity Score Matching			
Characteristic	Reduced Albumin (*n* = 94,925)	Normal Albumin (*n* = 146,303)	*p*	SMD	Reduced Albumin (*n* = 79,751)	Normal Albumin (*n* = 79,751)	*p*	SMD
**Demographics**								
Age at index, mean ± SD	64.0 ± 16.7	64.2 ± 16.9	0.005	0.012	64.3 ± 16.8	64.5 ± 17.0	0.003	0.015
White	58,717 (61.9%)	91,961 (62.9%)	0.006	0.012	50,297 (63.1%)	50,298 (63.1%)	0.996	<0.001
Male	51,402 (54.2%)	80,553 (55.1%)	0.012	0.011	43,596 (54.7%)	42,946 (53.9%)	0.001	0.016
Female	42,259 (44.5%)	64,814 (44.3%)	0.010	0.011	36,109 (45.3%)	36,747 (46.1%)	0.001	0.016
Black or African American	16,919 (17.8%)	23,798 (16.4%)	<0.001	0.045	13,902 (17.4%)	13,839 (17.4%)	0.677	0.002
Asian	5898 (6.3%)	11,473 (7.9%)	<0.001	0.062	5255 (6.6%)	4960 (6.2%)	0.003	0.015
**Diagnoses**								
Hypertensive diseases	60,191 (63.4%)	74,577 (51.0%)	<0.001	0.265	48,106 (60.3%)	49,218 (61.7%)	<0.001	0.029
Disorders of lipoprotein metabolism	39,023 (41.1%)	53,605 (36.6%)	<0.001	0.098	32,106 (40.3%)	32,520 (40.8%)	0.035	0.011
Diabetes mellitus	27,330 (28.8%)	30,510 (21.0%)	<0.001	0.190	21,281 (26.7%)	21,949 (27.5%)	0.000	0.019
Ischemic heart disease	27,155 (28.6%)	30,062 (20.5%)	<0.001	0.193	20,987 (26.3%)	21,573 (27.1%)	0.001	0.017
Atrial fibrillation and flutter	19,574 (20.6%)	20,721 (14.2%)	<0.001	0.175	15,023 (18.8%)	15,617 (19.6%)	0.000	0.019
Chronic kidney disease	19,411 (20.4%)	19,346 (13.3%)	<0.001	0.198	14,688 (18.4%)	15,205 (19.1%)	0.001	0.017
Heart failure	19,026 (20.0%)	18,104 (12.4%)	<0.001	0.214	14,172 (17.8%)	14,717 (18.5%)	0.000	0.018
Cerebral edema	22,444 (23.6%)	14,097 (9.7%)	<0.001	0.388	13,761 (17.3%)	13,750 (17.2%)	0.942	0.000
Obesity/overweight	16,977 (17.9%)	20,561 (14.1%)	<0.001	0.108	13,382 (16.8%)	13,542 (17.0%)	0.285	0.005
Other sepsis	14,410 (15.2%)	8854 (6.1%)	<0.001	0.304	8835 (11.1%)	8456 (10.6%)	0.002	0.015
Malnutrition	13,632 (14.5%)	8051 (5.5%)	<0.001	0.303	8275 (10.4%)	7745 (9.7%)	<0.001	0.022
Compression of the brain	12,816 (13.5%)	7275 (5.0%)	<0.001	0.302	7380 (9.3%)	7042 (8.8%)	0.003	0.015
Coma	11,126 (11.7%)	6984 (4.8%)	<0.001	0.258	6865 (8.6%)	6571 (8.2%)	0.008	0.013
GCS score, mean ± SD	12.3 ± 3.9	14.2 ± 2.3	<0.0001	0.588	13.1 ± 3.1	13.2 ± 3.1	0.284	0.032
Severe sepsis	7571 (8.1%)	3732 (2.6%)	<0.001	0.248	4202 (5.3%)	3696 (4.6%)	<0.001	0.029
Liver cirrhosis	4482 (4.8%)	2897 (2.0%)	<0.001	0.155	2908 (3.6%)	2763 (3.5%)	0.050	0.010
**Procedures**								
ECG ≥ 12 leads	51,139 (53.9%)	60,471 (41.3%)	<0.001	0.263	40,187 (50.4%)	41,332 (51.8%)	<0.001	0.029
Echocardiography	32,107 (33.8%)	34,893 (23.8%)	<0.001	0.228	24,547 (30.8%)	24,946 (31.3%)	0.031	0.011
Cardiac catheterization	6015 (6.4%)	7329 (5.0%)	<0.001	0.059	4801 (6.0%)	4925 (6.2%)	0.194	0.007
Craniectomy/craniotomy for ICH	1133 (1.2%)	523 (0.4%)	<0.001	0.096	589 (0.7%)	512 (0.6%)	0.020	0.012
Decompressive craniectomy or hematoma evacuation	8553 (9.0%)	4559 (3.1%)	<0.0001	0.247	4894 (6.1%)	4400 (5.5%)	<0.0001	0.026
**Medications**								
Beta blockers	50,167 (52.8%)	57,439 (39.3%)	<0.001	0.285	39,390 (49.4%)	40,684 (51.0%)	<0.001	0.033
Anticoagulants	46,471 (49.0%)	52,239 (35.7%)	<0.001	0.279	36,244 (45.4%)	37,425 (46.9%)	<0.001	0.030
Antiarrhythmics	45,801 (48.2%)	52,596 (36.0%)	<0.001	0.259	35,714 (44.8%)	36,473 (45.7%)	0.000	0.019
Calcium channel blockers	41,383 (43.6%)	46,228 (31.6%)	<0.001	0.257	32,054 (40.2%)	33,019 (41.4%)	<0.001	0.025
Diuretics	38,053 (40.1%)	42,273 (28.9%)	<0.001	0.244	29,320 (36.8%)	30,003 (37.6%)	0.000	0.018
Antilipemic agents	35,165 (37.0%)	47,843 (32.7%)	<0.001	0.097	28,943 (36.3%)	29,306 (36.7%)	0.059	0.010
Platelet aggregation inhibitors	30,832 (32.5%)	41,501 (28.5%)	<0.001	0.095	25,401 (31.9%)	25,703 (32.2%)	0.105	0.008
Antianginals	14,772 (15.6%)	18,634 (12.8%)	<0.001	0.085	11,993 (15.0%)	12,272 (15.4%)	0.052	0.010

Data are presented as *n* (%) for categorical variables and mean ± SD for continuous variables. Abbreviated for clarity; the full covariate set is provided in Supplementary Table S1. SMD, standardized mean difference (values < 0.10 indicate adequate balance after matching); ICH, intracerebral hemorrhage; ECG, electrocardiography; GCS, Glasgow Coma Scale. Before matching: reduced albumin *n* = 94,925; normal albumin *n* = 146,303. After 1:1 propensity score matching: *n* = 79,751 per group.

**Table 2:** Thirty-Day Clinical Outcomes After Propensity Score Matching.

Outcome	Reduced Albumin *n* (%) (*n* = 79,751)	Normal Albumin *n* (%) (*n* = 79,751)	Risk Difference (95% CI)	Risk Ratio (95% CI)	Hazard Ratio (95% CI)	*p*-Value
Composite outcome	32,233 (40.4%)	26,587 (33.3%)	7.08% (6.61, 7.55)	1.21 (1.20, 1.23)	1.28 (1.26, 1.30)	<0.0001
All-cause death	18,222 (22.8%)	12,793 (16.0%)	6.81% (6.42, 7.19)	1.42 (1.40, 1.45)	1.50 (1.46, 1.53)	<0.0001
Heart failure	9327 (11.7%)	7931 (9.9%)	1.75% (1.45, 2.05)	1.18 (1.14, 1.21)	1.22 (1.18, 1.26)	<0.0001
Myocardial infarction	3587 (4.5%)	2283 (2.9%)	1.64% (1.45, 1.82)	1.57 (1.49, 1.65)	1.36 (1.25, 1.47)	<0.0001
Atrial fibrillation/flutter	11,167 (14.0%)	11,206 (14.1%)	−0.05% (−0.39, 0.29)	1.00 (0.97, 1.02)	1.02 (0.99, 1.05)	0.779
Ventricular arrhythmia	1587 (2.0%)	1162 (1.5%)	0.53% (0.41, 0.66)	1.37 (1.27, 1.47)	1.41 (1.31, 1.52)	<0.0001
Takotsubo syndrome	234 (0.3%)	170 (0.2%)	0.08% (0.03, 0.13)	1.38 (1.13, 1.68)	1.41 (1.16, 1.72)	0.0014

Data are presented as *n* (%). Hazard ratios were derived from Cox proportional hazards regression in the 1:1 propensity-matched cohorts (*n* = 79,751 per group), with survival compared by the log-rank test; the *p*-value column reports the hazard ratio (log-rank) test. All outcomes were assessed within 30 days of the index intracerebral hemorrhage. CI, confidence interval.

## Data Availability

The data that support the findings of this study are available through the TriNetX US Collaborative Network (https://trinetx.com (accessed on 30 June 2026)). Access is subject to institutional membership and data use agreements with the TriNetX platform. Aggregated, de-identified data supporting the findings may be made available from the corresponding author upon reasonable request, subject to applicable data governance policies.

## Supplementary Materials

The following supporting information can be downloaded at: https://www.mdpi.com/article/10.3390/jcm15176840/s1, Table S1: ICD-10-CM Codes and Procedure Codes for All Outcomes, Exposures, and Propensity Score Matching Covariates; Table S2: Baseline Characteristics Before and After Propensity Score Matching—Sensitivity Analysis 5: Excluding Patients with Chronic Hypoalbuminemia; Table S3: Risk of Primary and Secondary Outcomes in the Sensitivity Analyses.

## References

[1] Feigin V.L., Brainin M., Norrving B., Martins S.O., Pandian J., Lindsay P., Grupper M.F., Rautalin I. (2024). World Stroke Organization (WSO): Global Intracerebral Hemorrhage Factsheet 2025. Int. J. Stroke.

[2] Wolsink A., Cliteur M.P., van Asch C.J., Boogaarts H.D., Dammers R., Hannink G., Schreuder F.H.B.M., Klijn C.J.M. (2024). Incidence, case fatality, and functional outcome of intracerebral haemorrhage, according to age, sex, and country income level: A systematic review and meta-analysis. Lancet Reg. Health Eur..

[3] Greenberg S.M., Ziai W.C., Cordonnier C., Dowlatshahi D., Francis B., Goldstein J.N., Hemphill J.C., Johnson R., Keigher K.M., Mack W.J. (2022). 2022 Guideline for the Management of Patients With Spontaneous Intracerebral Hemorrhage: A Guideline From the American Heart Association/American Stroke Association. Stroke.

[4] Li L., Murthy S.B. (2022). Cardiovascular Events After Intracerebral Hemorrhage. Stroke.

[5] Hoad K.L., Jones H., Miller G., Abdul-Rahim A.H., Lip G.Y., Buckley B.J. (2025). Stroke-heart syndrome: Incidence and clinical outcomes of cardiac complications following intracerebral haemorrhage. Eur. Stroke J..

[6] Fakhraei R., Song Y., Kazi D.S., Wadhera R.K., de Lemos J.A., Das S.R., Morrow D.A., Dahabreh I.J., Rutan C.M., Thomas K. (2025). Impact of Acute Myocardial Injury on Short- and Long-Term Outcomes in Patients With Primary Intracerebral Hemorrhage. J. Am. Heart Assoc..

[7] Scheitz J.F., Sposato L.A., Schulz-Menger J., Nolte C.H., Backs J., Endres M. (2022). Stroke-Heart Syndrome: Recent Advances and Challenges. J. Am. Heart Assoc..

[8] Du Y., Demillard L.J., Ren J. (2021). Catecholamine-induced cardiotoxicity: A critical element in the pathophysiology of stroke-induced heart injury. Life Sci..

[9] Chen Z., Venkat P., Seyfried D., Chopp M., Yan T., Chen J. (2017). Brain-Heart Interaction: Cardiac Complications After Stroke. Circ. Res..

[10] Li W., Li L., Chopp M., Venkat P., Zacharek A., Chen Z., Landschoot-Ward J., Yan T., Chen J. (2018). Intracerebral Hemorrhage Induces Cardiac Dysfunction in Mice Without Primary Cardiac Disease. Front. Neurol..

[11] Xu C., Zheng A., He T., Cao Z. (2020). Brain-Heart Axis and Biomarkers of Cardiac Damage and Dysfunction after Stroke: A Systematic Review and Meta-Analysis. Int. J. Mol. Sci..

[12] Arques S. (2020). Serum albumin and cardiovascular disease: State-of-the-art review. Ann. Cardiol. Angeiol..

[13] Chien S.C., Chen C.Y., Lin C.F., Yeh H.I. (2017). Critical appraisal of the role of serum albumin in cardiovascular disease. Biomark. Res..

[14] Biancucci M., Barbiero R., Pennella B., Cannatà A., Ageno W., Tangianu F., Maresca A.M., Dentali F., Bonaventura A. (2024). Hypoalbuminaemia and heart failure: A practical review of current evidence. Eur. J. Heart Fail..

[15] Morotti A., Marini S., Lena U.K., Crawford K., Schwab K., Kourkoulis C., Ayres A.M., Gurol M.E., Viswanathan A., Greenberg S.M. (2017). Significance of admission hypoalbuminemia in acute intracerebral hemorrhage. J. Neurol..

[16] Limaye K., Yang J.D., Hinduja A. (2016). Role of admission serum albumin levels in patients with intracerebral hemorrhage. Acta Neurol. Belg..

[17] Di Napoli M., Behrouz R., Topel C.H., Misra V., Pomero F., Giraudo A., Pennati P., Masotti L., Schreuder F.H.B.M., Staals J. (2017). Hypoalbuminemia, systemic inflammatory response syndrome, and functional outcome in intracerebral hemorrhage. J. Crit. Care.

[18] Boehme A.K., Comeau M.E., Langefeld C.D., Lord A., Moomaw C.J., Osborne J., James M.L., Martini S., Testai F.D., Woo D. (2018). Systemic inflammatory response syndrome, infection, and outcome in intracerebral hemorrhage. Neurol. Neuroimmunol. Neuroinflamm..

[19] Herrmann F.R., Safran C., Levkoff S.E., Minaker K.L. (1992). Serum albumin level on admission as a predictor of death, length of stay, and readmission. Arch. Intern. Med..

[20] Ishiguchi H., Huang B., El-Bouri W.K., Dawson J., Lip G.Y.H., Abdul-Rahim A.H., on behalf of the VISTA Collaborators (2024). Incidence and Outcomes of Patients with Early Cardiac Complications After Intracerebral Hemorrhage: A Report from VISTA. Stroke.

[21] Hess F., McGinnis J., Baki E., Wiltgen T., Müller A., Maegerlein C., Kirschke J., Zimmer C., Hemmer B., Wunderlich S. (2025). Predictors and Implications of Myocardial Injury in Intracerebral Hemorrhage. Clin. Neuroradiol..

[22] Wan J., Zhang S., Yuan X., Liu Y., Cheng X., Liu H., Yang F., Ai Y., Luo X., Zhong Y. (2025). Neutrophil-to-albumin ratio as a predictor of mortality in patients with intracerebral hemorrhage: A multicenter retrospective cohort study. BMC Neurol..

[23] Loggini A., Mansour A., El Ammar F., Tangonan R., Kramer C.L., Goldenberg F.D., Lazaridis C. (2021). Inversion of T Waves on Admission is Associated with Mortality in Spontaneous Intracerebral Hemorrhage. J. Stroke Cerebrovasc. Dis..

[24] Wang Q., Tu Y., Huang Y., Chen L., Lin Y., Zhan L., He J. (2022). High fibrinogen to albumin ratio is associated with hematoma enlargement in spontaneous intracerebral hemorrhage. J. Clin. Neurosci..

[25] Bucci T., Pastori D., Pignatelli P., Ntaios G., Abdul-Rahim A.H., Violi F., Lip G.Y.H. (2024). Albumin Levels and Risk of Early Cardiovascular Complications After Ischemic Stroke: A Propensity-Matched Analysis of a Global Federated Health Network. Stroke.

[26] Belinskaia D.A., Voronina P.A., Shmurak V.I., Jenkins R.O., Goncharov N.V. (2021). Serum albumin in health and disease: Esterase, antioxidant, transporting and signaling properties. Int. J. Mol. Sci..

[27] Arques S. (2018). Human serum albumin in cardiovascular diseases. Eur. J. Intern. Med..

[28] Putaala J., Lehto M., Meretoja A., Silvennoinen K., Curtze S., Kääriäinen J., Koivunen R.-J., Kaste M., Tatlisumak T., Strbian D. (2014). In-Hospital Cardiac Complications after Intracerebral Hemorrhage. Int. J. Stroke.

[29] Grimm G., Haslacher H., Kampitsch T., Endler G., Marsik C., Schickbauer T., Wagner O., Jilma B. (2009). Sex differences in the association between albumin and all-cause and vascular mortality. Eur. J. Clin. Investig..

[30] Cabrerizo S., Cuadras D., Gomez-Busto F., Artaza-Artabe I., Marin-Ciancas F., Malafarina V. (2015). Serum albumin and health in older people: Review and meta analysis. Maturitas.

[31] Loggini A., Saleh Velez F.G., Towner J.E., Hornik J., Wallery S.S., Battaglini D., Schwertman A., Nomani S., Hornik A., Qureshi A.I. (2025). Two decades of trends in nontraumatic intracerebral hemorrhage care: A nationwide analysis. J. Clin. Neurosci..

[32] Loggini A., Del Brutto V.J., El Ammar F., Bulwa Z.B., Saleh Velez F., McKoy C., Martinez R.C., Brorson J., Goldenberg F.D., Ardelt A.A. (2020). Intracranial Hemorrhage in Hospitalized Patients: An Infrequently Studied Condition with High Mortality. Neurocrit. Care.

[33] Tabata F., Wada Y., Kawakami S., Miyaji K. (2021). Serum Albumin Redox States: More Than Oxidative Stress Biomarker. Antioxidants.

[34] Kang K., Shi K., Liu J., Li N., Wu J., Zhao X. (2024). Autonomic dysfunction and treatment strategies in intracerebral hemorrhage. CNS Neurosci. Ther..

[35] Aldecoa C., Llau J.V., Nuvials X., Artigas A. (2020). Role of albumin in the preservation of endothelial glycocalyx integrity and the microcirculation: A review. Ann. Intensive Care.

[36] Bihari S., Bannard-Smith J., Bellomo R. (2020). Albumin as a drug: Its biological effects beyond volume expansion. Crit. Care Resusc..

[37] Violi F., Novella A., Pignatelli P., Castellani V., Tettamanti M., Mannucci P.M., Nobili A., on behalf of the REPOSI (REgistro POliterapie SIMI, Società Italiana di Medicina Interna) Study Group (2023). Low serum albumin is associated with mortality and arterial and venous ischemic events in acutely ill medical patients. Thromb. Res..

[38] Tian B., Tang X., He L., Cheng J., Wang J., Liang S., Mo J., Chen C. (2025). Predictors and Outcomes of Acute Kidney Injury in Intracerebral Hemorrhage Patients: Evidence from a Large-Scale National Database Analysis. Shock.

[39] Wiedermann C.J., Wiedermann W., Joannidis M. (2010). Hypoalbuminemia and acute kidney injury: A meta-analysis of observational clinical studies. Intensive Care Med..

[40] Irfan B., Ghafarian A.M., Salahie A., Shihabi K., Ewidat O., Riyad N., Kamel M., Khan I., Tesfaye B. (2026). Nutrition’s Impact on Recovery in Craniofacial and Otorhinolaryngological Injuries: Considering Areas of Armed Conflict and Humanitarian Emergencies. J. Craniofac. Surg..

[41] Loggini A., Saleh Velez F.G., Qureshi A.I., Ghafarian A., Wajahath M., Shahait A.D. (2026). Radiographic Stability to Guide Initiation of Pharmacologic Venous Thromboembolism Prophylaxis After Spontaneous Intracerebral Hemorrhage: A Systematic Review and Meta-Analysis. Neurohospitalist.

[42] Wilkes M.M., Navickis R.J. (2001). Patient Survival after Human Albumin Administration: A Meta-Analysis of Randomized, Controlled Trials. Ann. Intern. Med..

[43] Finfer S., Bellomo R., Boyce N., French J., Myburgh J., Norton R., on behalf of the SAFE Study Investigators (2004). A Comparison of Albumin and Saline for Fluid Resuscitation in the Intensive Care Unit. N. Engl. J. Med..

[44] Ginsberg M.D., Palesch Y.Y., Hill M.D., Martin R.H., Moy C.S., Barsan W.G., Waldman B.D., Tamariz D., Ryckborst K.J., on behalf of the ALIAS and Neurological Emergencies Treatment Trials (NETT) Investigators (2013). High-dose albumin treatment for acute ischaemic stroke (ALIAS) part 2: A randomised, double-blind, phase 3, placebo-controlled trial. Lancet Neurol..

[45] ClinicalTrials.gov (2023). Albumin for Intracerebral Hemorrhage Intervention (ACHIEVE).

