# Supplementary material for: Admission Serum Albumin as a Predictor of Cardiovascular Complications in Non-Traumatic Intracerebral Hemorrhage
Source: J Clin Med. 2026 Sep 3;15(17):6840. doi: 10.3390/jcm15176840 (PMC13565783; doi:10.3390/jcm15176840)
Supplement: Supplementary file 1 [file jcm-15-06840-s001.zip › jcm-4441342-supplementary.pdf]

Supplementary Tables

Table S1. ICD-10-CM Codes and Procedure Codes for All Outcomes, Exposures, and Propensity Score Matching Covariates

Outcome	ICD-10-CM Code(s)
**Primary Composite Outcome**	
All-cause death	Deceased (TriNetX mortality flag)
Acute myocardial infarction	I21
Heart failure	I50
Atrial fibrillation / flutter	I48
Ventricular Arrhythmia	I47.2, I49.0
Takotsubo syndrome	I51.81
**Index Event & Exposure**	
Intracerebral hemorrhage (index)	I61
Reduced albumin (exposed)	LOINC 1751-7 ≤3.4 g/dL
Normal albumin (reference)	LOINC 1751-7 ≥3.5 g/dL
**PSM Covariates - Diagnoses**	
Hypertensive diseases	I10-I16
Ischemic heart disease	I20-I25
Heart failure	I50
Atrial fibrillation	I48
Diabetes mellitus	E08-E13
Chronic kidney disease	N18
Dyslipidemia	E78
Obesity	E65-E68
Coma	R40.2, R40.20-R40.24
Cerebral edema	G93.6
Brain herniation	G93.5
Infratentorial ICH (cerebellar)	I61.3
Infratentorial ICH (brainstem)	I61.6
Pneumonia	J12-J18
Sepsis	A40-A41, R65.2
Liver cirrhosis	K74
Malnutrition	E40-E46
Nephrotic syndrome	N04
Peripheral arterial disease	I73.9
Prior hemorrhagic stroke	I61 (pre-index)
Pulmonary embolism	I26-I28
Crohn disease	K50
Ulcerative colitis	K51
Autoimmune / CTD	M30-M36
Burns	T20-T25
**PSM Covariates - Procedures**	
External ventricular drain (EVD)	CPT 61210; ICD-10-PCS 00H63YZ/00H64YZ

Outcome	ICD-10-CM Code(s)
Surgical decompression / craniotomy	CPT 61312, 61314, 61315
ECG ≥12 leads	CPT 93000-93010
Echocardiography	CPT 93303-93356
Cardiac catheterization	CPT 93451-93572
**PSM Covariates - Medications (ATC)**	
Anticoagulants	B01A
Antiplatelets	B01AC
Beta-blockers	C07
Antiarrhythmics	C01B
Diuretics	C03
Antilipemics	C10
Calcium channel blockers	C08
Antianginals	C01D

ATC = Anatomical Therapeutic Chemical classification. CPT = Current Procedural Terminology. LOINC = Logical Observation Identifiers Names and Codes. ICD-10-PCS = International Classification of Diseases, 10th Revision, Procedure Coding System.

Table S2. Baseline Characteristics Before and After Propensity Score Matching - Sensitivity Analysis 5: Excluding Patients with Chronic Hypoalbuminemia

	Before Propensity Score Matching				After Propensity Score Matching			
**Characteristic**	**Reduced Albumin (n = 47,666)**	**Normal Albumin (n = 95,475)**	**P-Value**	**SMD**	**Reduced Albumin (n = 43,159)**	**Normal Albumin (n = 43,159)**	**P-Value**	**SMD**
**Demographics**								
**Age, mean ± SD**	64.5 ± 17.1	63.9 ± 17.1	<0.0001	0.0312	64.6 ± 17.2	64.8 ± 17.2	0.0626	0.0127
White	29,450 (61.8%)	58,805 (61.6%)	0.0005	0.0196	27,230 (63.1%)	27,295 (63.2%)	0.6465	0.0031
Male	25,160 (52.8%)	52,933 (55.4%)	<0.0001	0.0409	23,298 (54.0%)	23,007 (53.3%)	0.0470	0.0135
Female	21,525 (45.1%)	41,707 (43.7%)	<0.0001	0.0410	19,818 (45.9%)	20,096 (46.6%)	0.0577	0.0129
Black or African American	7,641 (16.0%)	14,684 (15.4%)	<0.0001	0.0233	6,946 (16.1%)	6,890 (16.0%)	0.6034	0.0035
Asian	3,119 (6.5%)	8,266 (8.7%)	<0.0001	0.0770	2,954 (6.8%)	2,814 (6.5%)	0.0564	0.0130
Unknown Race	4,121 (8.6%)	7,612 (8.0%)	<0.0001	0.0282	3,824 (8.9%)	3,900 (9.0%)	0.3648	0.0062
Other Race	1,713 (3.6%)	4,086 (4.3%)	<0.0001	0.0331	1,581 (3.7%)	1,608 (3.7%)	0.6261	0.0033
**Diagnoses**								
Hypertensive diseases	27,364 (57.4%)	43,564 (45.6%)	<0.0001	0.2538	24,310 (56.3%)	24,735 (57.3%)	0.0035	0.0199
Disorders of lipoprotein metabolism	17,053 (35.8%)	30,897 (32.4%)	<0.0001	0.0816	15,543 (36.0%)	15,832 (36.7%)	0.0409	0.0139
Diabetes mellitus	11,466 (24.1%)	16,915 (17.7%)	<0.0001	0.1641	10,095 (23.4%)	10,467 (24.3%)	0.0030	0.0202
Ischemic heart disease	10,636 (22.3%)	15,756 (16.5%)	<0.0001	0.1546	9,347 (21.7%)	9,599 (22.2%)	0.0382	0.0141
Cerebral edema	10,841 (22.7%)	8,236 (8.6%)	<0.0001	0.4042	7,904 (18.3%)	7,664 (17.8%)	0.0336	0.0145
Atrial fibrillation and flutter	8,059 (16.9%)	11,068 (11.6%)	<0.0001	0.1587	7,037 (16.3%)	7,289 (16.9%)	0.0211	0.0157
Chronic kidney disease	6,966 (14.6%)	9,281 (9.7%)	<0.0001	0.1558	6,065 (14.1%)	6,357 (14.7%)	0.0046	0.0193
Heart failure	6,666 (14.0%)	8,462 (8.9%)	<0.0001	0.1671	5,732 (13.3%)	5,989 (13.9%)	0.0107	0.0174
Obesity / overweight	6,804 (14.3%)	11,342 (11.9%)	<0.0001	0.0764	6,093 (14.1%)	6,224 (14.4%)	0.2024	0.0087
Compression of brain	6,148 (12.9%)	4,144 (4.3%)	<0.0001	0.3144	4,175 (9.7%)	3,859 (8.9%)	0.0002	0.0252
Coma	5,313 (11.1%)	3,992 (4.2%)	<0.0001	0.2694	3,812 (8.8%)	3,566 (8.3%)	0.0027	0.0204

Chronic hypoalbuminemia exclusions applied: liver cirrhosis (K74), malnutrition (E40-E46), nephrotic syndrome (N04), Crohn disease (K50), ulcerative colitis (K51), autoimmune/CTD (M30-M36), burns (T20-T25), sepsis (A40-A41/R65.2), and documented normal albumin ≥3.5 g/dL at least 1 month prior to index date. Before matching: <3.4 g/dL n = 47,666; >3.5 g/dL n = 95,475. After matching: n = 43,159 per group. SMD = standardized mean difference.

Table S3. Risk of Primary and Secondary Outcomes in the Sensitivity Analyses

	Reduced albumin events, n (%)	Normal albumin events, n (%)	HR (95% CI)
**Patients aged >65 years n = 36,374 (each group)**			
Composite outcome	14,708 (40.4%)	12,998 (35.7%)	1.181 (1.150-1.213)
All-cause death	8,218 (22.6%)	6,190 (17.0%)	1.385 (1.340-1.432)
Heart failure	4,202 (11.6%)	3,717 (10.2%)	1.167 (1.117-1.220)
Myocardial Infarction	1,506 (4.1%)	1,041 (2.9%)	1.446 (1.323-1.569)
Atrial fibrillation and flutter	6,202 (17.1%)	6,542 (18.0%)	0.965 (0.932-0.999)
Ventricular Arrhythmia	651 (1.7%)	531 (1.4%)	1.24 (1.10–1.40)
Takotsubo syndrome	95 (0.3%)	71 (0.2%)	1.370 (1.008-1.864)
**Females only n = 36,134 (each group)**			
Composite outcome	14,357 (39.7%)	11,714 (32.4%)	1.296 (1.265-1.328)
All-cause death	8,147 (22.5%)	6,001 (16.6%)	1.419 (1.372-1.467)
Heart failure	4,060 (11.2%)	3,292 (9.1%)	1.278 (1.220-1.338)
Myocardial Infarction	1,642 (4.5%)	1,034 (2.9%)	1.587 (1.463-1.711)
Atrial fibrillation and flutter	4,865 (13.5%)	4,674 (12.9%)	1.065 (1.023-1.109)
Ventricular arrhythmias	548 (1.5%)	385 (1.1%)	1.463 (1.284-1.667)
Takotsubo syndrome	173 (0.5%)	126 (0.3%)	1.406 (1.118-1.769)
**Patients with multimorbidity n = 37,888 (each group)**			
Composite outcome	16,058 (42.4%)	13,866 (36.6%)	1.214 (1.187-1.242)
All-cause death	7,648 (20.2%)	5,666 (15.0%)	1.402 (1.355-1.451)
Heart failure	5,244 (13.8%)	4,647 (12.3%)	1.162 (1.117-1.209)
Myocardial Infarction	1,613 (4.3%)	1,085 (2.9%)	1.487 (1.378-1.603)
Atrial fibrillation and flutter	6,494 (17.1%)	6,556 (17.3%)	1.010 (0.976-1.045)
Ventricular Arrhythmias	748 (2.0%)	630 (1.7%)	1.219 (1.097-1.355)
Takotsubo syndrome	86 (0.2%)	90 (0.2%)	0.975 (0.725-1.310)
**Mild reduced albumin (2.8-3.4 g/dL) n = 44,705 (each group)**			
Composite outcome	16,543 (37.0%)	14,694 (32.9%)	1.161 (1.136-1.187)
All-cause death	9,034 (20.2%)	6,851 (15.3%)	1.368 (1.331-1.405)
Heart failure	4,846 (10.8%)	4,399 (9.8%)	1.131 (1.092-1.172)
Myocardial Infarction	1,820 (4.1%)	1,280 (2.9%)	1.422 (1.311-1.533)
Atrial fibrillation and flutter	5,951 (13.3%)	6,246 (14.0%)	0.968 (0.939-0.999)
Ventricular Arrhythmias	790 (1.8%)	649 (1.5%)	1.251 (1.143-1.368)
Takotsubo syndrome	117 (0.3%)	92 (0.2%)	1.307 (1.031-1.656)
**Severely reduced albumin (≤2.7 g/dL) n = 27,392 (each group)**			
Composite outcome	12,393 (45.2%)	9,179 (33.5%)	1.494 (1.454-1.535)
All-cause death	7,752 (28.3%)	4,392 (16.0%)	1.947 (1.876-2.021)
Heart failure	3,488 (12.7%)	2,964 (10.8%)	1.248 (1.188-1.311)
Myocardial Infarction	1,434 (5.2%)	784 (2.9%)	1.829 (1.687-1.971)
Atrial fibrillation and flutter	3,718 (13.6%)	3,791 (13.8%)	1.026 (0.980-1.073)
Ventricular Arrhythmias	803 (2.9%)	511 (1.9%)	1.495 (1.413-1.799)
Takotsubo syndrome	99 (0.4%)	64 (0.2%)	1.599 (1.167-2.192)
**Excluding chronic hypoalbuminemia n = 43,159 (each group)**			
Composite outcome	14,935 (34.6%)	12,465 (28.9%)	1.261 (1.232-1.292)
All-cause death	8,556 (19.8%)	6,055 (14.0%)	1.486 (1.437-1.537)

	Reduced albumin events, n (%)	Normal albumin events, n (%)	HR (95% CI)
Heart failure	3,587 (8.3%)	3,264 (7.6%)	1.142 (1.088-1.199)
Myocardial Infarction	1,908 (4.4%)	1,236 (2.9%)	1.544 (1.431-1.657)
Atrial fibrillation and flutter	4,930 (11.4%)	5,416 (12.5%)	0.934 (0.898-0.971)
Ventricular Arrhythmias	552 (1.3%)	437 (1.0%)	1.309 (1.151-1.489)
Takotsubo syndrome	84 (0.2%)	67 (0.2%)	1.309 (0.943-1.817)

HR: Hazard Ratio; CI: Confidence Interval. All HRs derived from Kaplan-Meier survival analysis with log-rank testing. Data presented as n (%) with percentages rounded to one decimal place. Ventricular arrhythmias includes ventricular tachycardia (I47.2) and ventricular fibrillation (I49.0). Each sensitivity analysis used separate 1:1 propensity score matching; n shown reflects matched pairs per group.
